# Nanopesticides in Brazilian Crops: Classes, Mechanisms, Efficacy, Risks, and Photocatalytic Remediation

**DOI:** 10.3390/plants14182880

**Published:** 2025-09-16

**Authors:** Tatiana Cardoso e Bufalo, Victor Hugo Buttrós, Aline Bastos de Paiva, Deyne Dehon de Oliveira, Caio Silas Ferreira Ribeiro, Joyce Dória

**Affiliations:** 1 Physics Graduate Program, Department of Physics, Natural Science Institute, Federal University of Lavras (UFLA), Lavras 37203-202, MG, Brazil; alinepaiva@ufla.br (A.B.d.P.); deyne.oliveira1@estudante.ufla.br (D.D.d.O.); 2 Agricultural Microbiology Graduate Program, Department of Biology, Natural Science Institute, Federal University of Lavras (UFLA), Lavras 37203-202, MG, Brazil; victor.buttros1@ufla.br (V.H.B.); caio.ribeiro3@estudante.ufla.br (C.S.F.R.); 3 Department of Agriculture, Federal University of Lavras (UFLA), Lavras 37203-202, MG, Brazil

**Keywords:** agribusiness, agrochemicals, nanotechnology, nanoremediation

## Abstract

Brazil leads tropical agriculture, yet annual yield losses from insect pests and concerns over water contamination, non-target impacts, and resistance sustain demand for safer, more efficient control tools. This review synthesizes advances in nanopesticides for Brazil’s major crops (soybean, sugarcane, coffee, and citrus) and is organized into five parts, comprising concepts and definitions; formulation families; modes of action; efficacy evidence from laboratory, greenhouse, and field studies; and environmental and human health risk considerations. Evidence indicates that nano-enabled delivery can increase on-target deposition, prolong residual activity, and match or exceed control at reduced active ingredient loads by improving foliar adhesion, transcuticular transport, plant uptake, and spatiotemporal targeting with pheromone-releasing nanofibers and other dispensers. Because nanoformulations can alter exposure pathways and environmental fate, this review emphasizes nano-specific physicochemical characterization under use conditions, fate and transport in Oxisols and Ultisols, and tropical waters, ecotoxicity panels that include pollinators, aquatic invertebrates, soil biota, and vertebrate surrogates, and scenario-based exposure assessment for applicators, residents, and consumers. The review closes with practical guidance for Brazil: head-to-head efficacy benchmarks against commercial standards, the standardized reporting of release and characterization data, and a nano-specific environmental risk assessment checklist to help realize efficacy gains while protecting environmental and human health.

## 1. Introduction

Brazil stands out as the world’s largest producer and exporter of food, wherein the agribusiness sector accounts for about 25% of the national gross domestic product [1], presenting successive record harvests of production in the countryside, accounting for the largest surplus ever recorded in Brazilian history at USD 141.8 billion in 2022 [2]. As a consequence, there was an expectation of an increase in the area to be harvested in 2023 of 4.6% concerning the extent of cultivated fields compared to 2022, corresponding to 3.4 million hectares [3], compelling Brazilian producers to sustainably manage lands [4] and adopt new technologies [5] to improve crop yield, a strategy also protecting Brazilian biomes from deforestation [6,7,8]. Intrinsic to resources generated by agriculture, insect pest outbreaks are favored by monocultures in extensive areas due to the lack of important ecosystem services that control insect pest populations, causing severe production losses in crops [9,10].

Agrochemicals encompass chemical products used as pesticides, considered essential in large-scale agricultural production [11]. The growing demand for food promotes the consumption of insecticides, despite the environmental and human health risks and the development of resistance of target populations to pesticides [12]. Brazilian agricultural policy allows agricultural financing for land or machinery acquisition, conditioned on the buying of pesticides, also motivated by alleged reduced labor costs in agricultural pest control, making Brazil the world’s largest consumer of pesticides [13,14]. The abuse of insecticides in Brazil has the potential to compromise food security globally, encouraging efforts to gradually replace current pesticides with healthful products or practices [15]. Indeed, strong evidence on pesticide poisoning in Brazil dates from the early 1970s [16,17,18,19,20,21,22,23,24,25,26,27,28,29,30,31,32].

The evolution of technologies in pesticides, from inorganic products and industrial by-products (1850–1940) to synthetic organic compounds (1940–1970) and lower-risk synthetic organic compounds (1970–present) [33], realizes the search for sustainable agriculture, comprising the understanding of the complexity of agricultural systems and the re-designing of agricultural practices that minimize harmful impacts on ecosystems without jeopardizing the food security and general welfare of future generations [34]. This scenario allows for the emergence of nanotechnology as an innovative and sustainable strategy to enhance crop protection and nutrition, detect plant pathogens, increase plant resilience to environmental factors, improve soil structure and health, and aid crop biofortification [35]. Nanomaterials in pesticide formulations are premised on the precise delivery of agrochemicals to the target, as well as the improvement of the dispersion and stability of active ingredients, the lowering of residual pollution, and a reduction in labor costs [35]. Engineered nanomaterials for targeted pesticides in an environmentally responsive controlled release offer great potential for creating safe nanoformulations [36]. The high surface-to-volume ratio characteristic of nanostructures makes them more efficient in comparison with their bulk components, and the low dose released over a prolonged period is supposed to reduce the excess run-off of unwanted pesticides [37]. The mechanisms of action of nanopesticides comprise external damage to pests by acting on the integrity of the cuticle and internal impairment of insect development via the disruption of nutrient intake and altered biochemical activity, such as a rise in reactive oxygen species (ROS) and pro-inflammatory mediators, which also damage the reproduction of plagues [38].

Despite the alleged eco-friendly approach of nanoagrochemicals for sustainable agriculture, as its bulk counterpart (conventional agrochemicals), nanopesticides also present risks for human and environment health via the deposition of nanomaterials in nature, such as in water resources and soil, as well as residues in food products, with the disturbing fact of being potentially hazardous even in very low concentrations/doses due to the direct and intentional application in the environment [39,40]. Considering the lack of a clear definition of nanopesticides for regulatory purposes, and the unique characteristics of nanomaterials against their bulk counterpart, standard ecotoxicity testing may not apply to nanoagrochemicals [41]. Therefore, in countries where the economy is heavily dependent on the agricultural trade, as is the case in Brazil, farmers and residents could potentially be exposed to the poisonous effects of nanoagrochemicals.

## 2. Nanopesticide Concepts, Formulations, Efficacy, and Modes of Action

**Figure 1:**
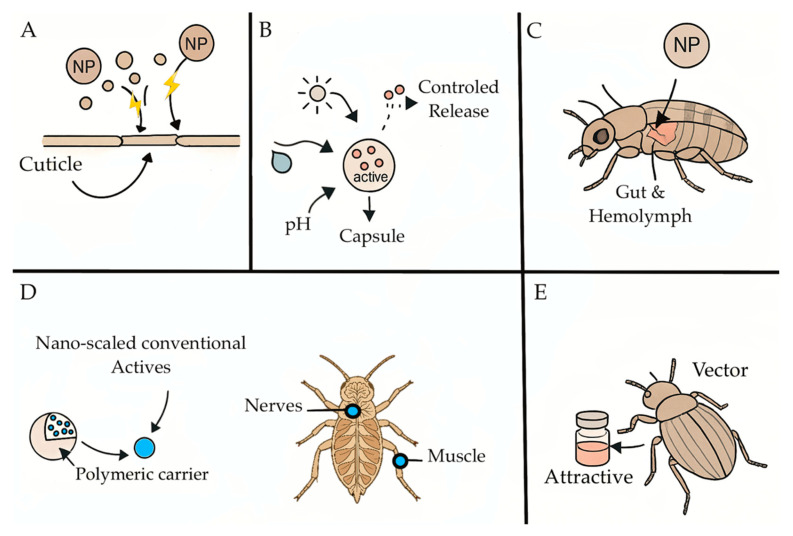
Illustration of the five major modes of action in nanopesticides. (**A**) External disruption of cuticles and membranes; (**B**) stimulus-responsive release (pH, enzymes, and light); (**C**) enhanced uptake to the gut/hemolymph; (**D**) interference with neuromuscular targets carrying conventional actives; and (**E**) attract-and-kill or dispenser systems to vectors.

Nanopesticides are pesticides with active or biocidal functionalities carried or enabled by nanoscale structures designed to improve delivery, stability, and target specificity [41,42,43]. The most common formulation families include (I) polymeric nanocarriers (e.g., chitosan, PLGA, and star polycations) for controlled/triggered release [44,45,46]; (II) inorganic nanomaterials (e.g., metal oxides, metal/metal–oxide composites) with intrinsic bioactivity [47,48,49]; (III) nanoemulsions/liposomes improving dispersion and cuticular penetration [46,50,51]; and (IV) hybrid or “green-synthesized” nanoparticles using plant/microbial reductants for potentially improved biocompatibility [52,53,54,55]. Modes of action are illustrated on Figure 1, and include (A) the external disruption of cuticles and membranes [47,48]; (B) stimulus-responsive release (pH, enzymes, and light), aligning pesticide flux with pest exposure windows [56,57,58,59,60]; (C) enhanced uptake to the gut/hemolymph, leading to oxidative stress and metabolic disturbance [45,47]; (D) interference with neuromuscular targets (e.g., AChE inhibition, ion channel modulation) when carrying conventional actives [43,61]; and (E) attract-and-kill or dispenser systems that physically position the active ingredient where vectors are present [42,43].

These mechanisms complement conventional active ingredients by improving spatial–temporal delivery while potentially lowering application rates, but they also introduce nano-specific exposure pathways that require a tailored risk assessment [41,42,43,44].

In efficacy terms, nano-enablement most often improves how and where the active reaches the pest rather than changing its molecular target, yielding practical gains such as higher control at equal or lower doses, longer residuals, or better performance under wash-off and UV stress [35,44,62]. Polymeric carriers illustrate this clearly: cationic star polymers (SPc) increase foliar wetting/retention and plant uptake, translating into stronger contact and the plant-uptake-dependent stomach toxicity of thiamethoxam against aphids compared with the non-nano reference [45,63]. Solid nanodispersions preserve crystallinity while improving the dispersion of poorly soluble actives; chlorantraniliprole nanodispersions maintained biological activity after high-pressure homogenization, supporting potency retention through processing and storage [64]. Field-scale translation is emerging: a unimolecule delivery system improved pest control across multiple pathosystems under farm conditions, consistent with enhanced penetration/deposition from the nano-architecture [50]. For citrus vector management, a thiamethoxam nanoformulation has been explored as an environmentally oriented option against *Diaphorina citri*, illustrating how delivery optimization can support efficacy goals within IPM programs [65].

Efficacy gains are also reported when carriers themselves contribute to bioactivity or when nanoscale properties intensify exposure at biological interfaces. Protein-based zein nanoparticles showed direct insecticidal effects against *Anticarsia gemmatalis*, indicating that some carriers add to overall potency beyond the encapsulation of a conventional toxicant [66]. Inorganic nanomaterials such as ZnO- and Cu-based nanoparticles provide intrinsic pesticidal activity via membrane disruption and oxidative stress, with demonstrated larvicidal and antifeedant effects against *Spodoptera frugiperda* in laboratory assays [47,48,67,68]. Reports of NP impacts on other agricultural pests (e.g., coffee berry borer) reinforce that intrinsic nano–bio interactions can be leveraged as tools. However, they should be benchmarked against registered standards and screened for crop safety [69,70].

Formulations that improve spreading and penetration also enhance apparent use efficiency in botanically based systems. Plant oil nanoemulsions and related nanoemulsion technologies increase stability and leaf coverage, supporting practical control outcomes in more sustainable programs and sometimes enabling dose reductions relative to coarse emulsions [55,71]. Temperature- or light-responsive and pH–enzyme-triggered systems further synchronize release with pest activity or the microenvironment, improving the fraction of actives delivered during biologically relevant windows [56,57,72,73]. Spatial targeting via nano-enabled dispensers can likewise boost operational efficacy without increasing toxicity per se: electrospun fibers and functional nano-dispensers sustain pheromone or semiochemical release for monitoring, mass trapping, or attract-and-kill, thereby increasing encounter rates between pests and actives in the field [58,59,60,74,75].

When reporting efficacy for nanoformulations, it is essential to include head-to-head comparisons with labeled commercial references at equal active loads, standardized endpoints (e.g., LC50/LC90, Abbott-corrected mortality, and field percent reduction with confidence intervals), and release/physicochemical data under use conditions (size, charge, dissolution, release kinetics) so that performance can be interpreted within exposure science and regulatory frameworks [42,49,76,77]. Notably, capsule chemistry can shift toxicity independent of size class; for example, differences between micro- and nano-capsules have been observed to depend on capsule composition, underscoring the need for case-by-case evaluation when claiming “dose reduction” benefits [78,79,80]. Integrating such nano-specific characterization with efficacy endpoints will allow robust comparisons across polymeric, inorganic, and hybrid platforms while maintaining alignment with environmental fate and risk assessment considerations already highlighted above [41,43,81]. Table 1 summarizes the formulations, effects, and proposed mechanisms of nanopesticides.

## 3. Nanotechnology in Coffee Crops

Brazil, the world’s largest coffee producer, had an area destined for coffee crops of 2.26 million hectares in 2023, resulting in exports reaching USD 9.2 billion [88]. During the coffee production cycle, biotic factors such as insect pests compromise productivity, resulting in significant economic losses. The country’s major coffee plants are the coffee berry borer *Hypothenemus hampei* (Coleoptera: Scolytidae) and the coffee leaf miner *Leucoptera coffeella* (Lepidoptera: Lyonetiidae) [89,90].

The coffee berry borer is an exotic species introduced to Brazil in 1913 from coffee seeds imported from the Democratic Republic of Congo, although the first official report dates from 1924 [91]. The difficulty in controlling the pest lies in the damage caused by its larvae that reside and feed inside coffee fruits, exposing the fruits to the action of opportunistic microorganisms, causing losses of up to 21% [92,93]. Its control was largely carried out by insecticides/acaricides containing the active ingredient endosulfan, banned in Brazil since 31 July 2013 [94]. The ban stems from the high environmental persistence and dangerousness of the pesticide, in addition to its relationship with human hormonal disorders and cancer incidence [95].

The persistent contamination of the environment by the organochlorine pesticide endosulfan, possessing a half-life period of 0.6 to 9 years, highly motivates a nanotechnological method to remedy it. Photocatalytic nanomaterials are capable of degrading hazardous organic compounds into less toxic/non-toxic intermediate/final products by solar light absorption at ambient temperature and normal atmospheric pressure [96]. Further, in a complementary approach, the feasibility of controlling coffee berry borer with nanoparticles was carried out by Brazilian researchers [69], pointing to a sublethal effect on the digestion of an artificial diet contaminated with zinc oxide (ZnO), copper oxide (CuO), and cerium oxide (${CeO}_{2}$) nanoparticles.

Coffee leaf miner is also an exotic species originating from the African continent, initially reported in 1842 on coffee plantations in the Caribbean Antilles and introduced to Brazil in 1850 [97]. Damage to coffee crops is also caused by its larvae, causing losses of 30–70% in the quality and production of grains [98]. In Brazil, chemical control by the organophosphate chlorpyrifos still prevails, a product that remains among the ten bestselling agrochemicals in Brazil, with a sales volume of 8.86 thousand tons in 2020, according to official data from the Brazilian government compiled by the Brazilian Institute For Environment and Natural Renewable Resources (*Instituto Brasileiro de Meio Ambiente e dos Recursos Naturais Renováveis*—*IBAMA*) [99]. The insecticide chlorpyrifos was banned in the USA, the European Union, and Argentina in 2021 due to severe damage to human health, including children’s neurodevelopment, even at levels below toxicity guidelines [100,101].

Chlorpyrifos is a highly toxic component that is found in natural water, crops, and soil due to its durability in water. Nanostructured photocatalysts based on titanium dioxide (${TiO}_{2}$) nanoparticles are capable of degrading chlorpyrifos using ultraviolet light (UV light), high-energy radiation, motivating the search for alternative photocatalytic materials able to degrade it with visible light [102]. Recently, copper nanoparticles under natural daylight accomplished the degradation of chlorpyrifos [103,104].

## 4. Nanotechnology in Sugarcane Crops

Brazil is the largest sugarcane producer in the world, producing 654.5 million tons in the 2020/2021 harvest, destined for the production of 41.2 million tons of sugar and 29.7 billion liters of ethanol [105]. The harvested area destined for sugar and alcohol activities in 2022/2023 corresponded to 8.1 million hectares [105]. Unfortunately, the magnitude of productivity in Brazilian sugarcane fields does not imply the extinction of their pests, which can cause equally gigantic losses. The main pests of sugarcane crops are the sugarcane borer *Diatraea saccharalis* (Lepidoptera: Pyralidae) [106], present throughout Brazil, affecting sugarcane productivity and the quality of sugar and alcohol production, with an estimated loss of around USD 1 billion for the 2022/2023 harvest, and the sugarcane weevil *Sphenophorus levis* (Coleoptera: Curculionidae) [107], an insect with complex management, present in more than 60% of sugarcane fields in the center–south of Brazil, which can generate losses of up to 25 tons per hectare and a reduction in the longevity of the crops (CTC 2023).

The sugarcane borer probably originates in South and Central America and is found throughout the Western Hemisphere, from the United States to Argentina. It is a difficult pest to control due to its extended stay inside the culm, opening galleries to feed, and favoring the lodgment of microorganisms such as fungi and bacteria [108]. Novaluron, a growth regulator belonging to the benzoylphenylurea insecticide class [109], not approved in the European Community (according to 2001/861/EC) [110], is purportedly a low-risk chemical, even though several researchers have reported that it has a hazardous effect on mammals, also exhibiting perilous effects on human male reproduction [111]. Regardless of its hazardous effects, nanoparticles of novaluron were synthesized, presenting bioactivity and toxicity similar to commercial (bulk) formulations, motivated by an alleged environmental benefit of agricultural systems due to its application in smaller quantities [84]. Seen from another perspective, photodegradation is a promising method for the removal of organic pollutants from water, owing to its high efficiency, environmental friendliness, low cost, and low secondary pollution. Hence, a carbon nitride nanofilm excited by visible light was able to degrade 64% of novaluron after two hours of Xenon-light irradiation [112].

The *Sphenophorus levis* genus originates from North America, and its presence in Brazilian sugarcane fields was first reported in Santa Bárbara D’Oeste, in the state of São Paulo, in 1977, having been described as a new species in 1978 [113]. The larval stage is responsible for crop damage, making it hard to control, since it can remain in the sugarcane field even after the cut of the ratoon. Despite strong chemical control, there has still been an increase in pest populations, with record numbers of new infested areas being frequent in recent years [114]. Thiamethoxam is widely used in sugarcane cultivation as a pesticide to control sucking insects and some chewing species, including the sugarcane weevil [115]. Besides its efficiency in pest control, thiamethoxam also stimulates sugarcane stalk productivity [116]. As a bioactivator, thiamethoxam increases energy cogeneration from sugarcane via higher biomass production [117].

Thiamethoxam is a second-generation neonicotinoid insecticide that has a level III toxicological classification (medium toxicity) and environmental class III classification (dangerous for the environment), causing acute kidney injury by direct toxicity in human beings [118] and decreasing homing success in honeybees, also impairing the physical ability of bees to fly [119,120]. The European regulatory body banned the use of thiamethoxam in open areas due to damage to bees, and, in 2018, while IBAMA, the Brazilian Institute of Environment and Renewable Natural Resources, indicated the suspension of its dispersion by aircraft for the same reason, this was immediately suspended due to economic reasons [110].

To improve the efficiency of conventional thiamethoxam and avoid excessive application and environmental pollution, researchers have developed its nanometerization using polymers and polymeric materials. A star polycation, when complexed with thiamethoxam, forms a complex that decreases the particle size of thiamethoxam to the nanoscale. Nano-sized thiamethoxam/star polycation complexes presented enhanced contact and stomach toxicity against green peach aphids [63]. It is worth highlighting that the impacts of star polycation nanocarriers on animal development and health, especially the underlying molecular mechanisms, are not fully understood. The determination of the biotoxicity of a widely applied star polycation nanocarrier using *Drosophila melanogaster* demonstrated its multiple levels of detrimental effects. Chronic exposure at sublethal-level concentrations showed long-lasting adverse effects on longevity, reproduction, and motor activity, providing a reference for understanding the hazards of star polycation nanocarriers and for developing guidelines for large-scale applications in crop fields [121].

On the other hand, thiamethoxam can be removed from water using metal hexacyanoferrate nanoparticles [122]. Highly crystalline nanoparticles of metal hexacyanoferrates of Zn, Cu, Co, and Ni were evaluated for the solar photocatalytic degradation of the hazardous pesticide thiamethoxam, reaching a maximum degradation extent of 70–98% [123]. Furthermore, thiamethoxam can also be removed from water and sugarcane juice by magnetic nanomodified activated carbon [124].

## 5. Nanotechnology in Orange Crops

Brazil has consolidated itself as the largest exporter of oranges and/or orange juice worldwide, while also being the second largest producer of citrus in the world. Orange production has become one of the most important agricultural activities in Brazil: in 2022, the value of orange production reached USD 3 billion, harvested from an area of 600 thousand hectares [125].

In the 2022/2023 harvest, citrus greening disease showed intense growth, with an average incidence of diseased trees of 24.42%, mainly due to favorable weather and the high density of citrus trees, supporting the spread of the Asian citrus psyllid *Diaphorina Citri* (Hemiptera: Psylloidea: Liviidae), the vector of the bacteria that causes greening disease in the groves [126], the worst disease in the citrus industry [127]. The fruit fly, especially the Mediterranean fruit fly, or Medfly, *Ceratitis capitata* (Diptera: Tephritidae), also remains a relevant pest in citrus farming, causing losses in the productivity of orchards that can reach between 30 and 50%. The insect larvae develop inside the fruits and feed on the pulp, making consumption unviable [128].

The Asian citrus psyllid is native to the Indian subcontinent, first recorded in Brazil in 1942, at that time considered a secondary pest until the first detection of citrus greening disease in 2004, the most feared disease among orange producers as it does not have any type of cure or treatment, when the insect assumed greater importance due to the transmission of bacteria associated with citrus greening disease [129,130]. As there is no efficient control of the disease, its management relies on the control of the insect vector of the bacteria.

Chemical control of the Asian citrus psyllid can be carried out with the now well-known thiamethoxam, including a purportedly environmentally friendly formulation based on nano-sized thiamethoxam [65,82]. In Brazil, in addition to thiamethoxam, the neonicotinoid imidacloprid has been one of the most used insecticides to control the *Diaphorina Citri*, also finding a functional nano-dispenser strategy to deliver imidacloprid in order to decrease the negative environmental impact of this widely used insecticide [74], besides the development of imidacloprid nanoparticles for an alleged environmental purpose [131]. Even though animal studies indicate the relatively low toxicity of imidacloprid to mammals, human poisoning leading to death has been reported [132,133,134]. Imidacloprid was the seventh best-selling insecticide in Brazil in 2019 [99]. The European Commission extended the ban of imidacloprid and thiamethoxam to all field crops due to strong evidence that the pesticides can harm domesticated honeybees and also wild pollinators [135]. Approaches for the elimination of imidacloprid from the environment, including physical (adsorption), chemical (oxidation, hydrolysis, and photodegradation), and biological (microbial degradation) remediation, have been the focus of intense research [136], which also comprises photodegradation by nanocomposites [137].

The Mediterranean fruit fly, one of the world’s most destructive fruit pests, originated in sub-Saharan Africa and was recorded for the first time in Brazilian territory in 1901 [138]. The economic losses caused by the Medfly encompass direct damage to citrus production and the quarantine barriers imposed by importing countries to contain its dissemination [139].

The chemical strategy to control the Medfly is based on monitoring by trimedlure-baited traps, a so-called environmentally friendly approach aiming to avoid the systematic application of conventional insecticides, feared due to the development of pesticide resistance and unwanted environmental effects. Trimedlure is a parapheromone that attracts males primarily and is only weakly attractive to females, having a simple chemical structure that allows relatively cheap production [140,141]. Trimedlure is banned in the EU and deemed to be obsolete [142,143]. Notwithstanding its prohibition in the EU, trimedlure remains an active ingredient with authorized use in Brazil [144], inspiring Brazilian researchers to develop nanofiber formulations containing trimedlure for the Medfly [75] as well as an alternative trimedlure-free formulation based on magnetite nanoparticles, resulting in larval toxicity expressed as dose-dependent lethality [83].

## 6. Nanotechnology in Soybean Crops

Brazil leads the ranking of the world’s largest soybean producers and exporters, with 156 million tons of grain produced in the 2022/2023 harvest, accounting for 42% of the world’s total soybean production, across a cultivated area of 44,062.6 million hectares. Currently, soy is the main export item for Brazilian agribusiness [145], requiring the protection of plantations against pests to ensure plant productivity. The fall armyworm *Spodoptera frugiperda* (Lepidoptera: Noctuidae) and the velvetbean caterpillar *Anticarsia gemmatalis* (Lepidoptera: Noctuidae) are considered the main defoliating pests of soybean, generating an average loss of up to 7.7% of grain production [146,147].

The fall armyworm was described in Brazil in 1953, and has its origins in North America [148]. Its extremely polyphagous and multi-host nature justify its difficult control, resulting from the reduction in its natural enemies due to the unrestrained use of broad-spectrum insecticides, in addition to acquired resistance to several agrochemicals. Possible insecticide options, although not registered for the control of *Spodoptera* caterpillars in Brazil, include thiodicarb, methomyl, chlorantraniliprole, and flubendiamide [149,150]. Thiodicarb is prohibited in the EU (Regulation (EC) No 1107/2009), methomyl is banned in China, the United Kingdom, Turkey, India, and the EU, chlorantraniliprole is banned for cosmetic use in Canada, and flubendiamide is banned in the US and the EU. Chlorantraniliprole causes long-lasting locomotor deficits in honeybees, as well as brain and muscular calcium channel alterations [151].

Regarding the application of nanoparticles against the fall armyworm, Cu, KI, Ag, and Bd nanoparticles against fourth-instar *Spodoptera frugiperda*, tested at three concentrations (1000, 10,000, and 100,000 ppm), show that nanoparticles can cause toxic effects on *S. frugiperda* larvae [85]. Commercial zinc oxide nanoparticles against the fall armyworm under laboratory conditions showed their potential to significantly reduce its population in the ecosystem through body deformations and reduced fecundity, oviposition, and hatchability of eggs [67]. Copper oxide nanoparticles presented remarkable larvicidal antifeedant activity against *Spodoptera* caterpillars [68]. Chlorantraniliprole nanoparticles were developed, claiming to be an environmentally friendly formulation by reducing both residues in food and environmental pollution caused by the pesticide [64]. An invention providing an alleged environmentally benign functionalized thiodicarb nanoparticle was claimed in patent WO2014164418A1 [152].

The velvetbean caterpillar is a species of neotropical origin, first recorded in the north of the USA in 1893 [143] and in Brazil in 1973 [153], considered the main defoliator in the Americas and one of the most common species in soybean cultures [154]. In Brazil, caterpillar control is predominantly carried out by spraying insecticides [155]. Currently, insecticides based on benzoylphenylureas, such as novaluron and teflubenzuron [146], and pyrrole insecticides, such as chlorfenapyr [156], have been the most widely used. Novaluron, teflubenzuron, and chlorfenapyr are not approved in the USA or EU [157]. Indeed, teflubenzuron figured in the list of the most frequently used active ingredients in the period of 2012–2016 in Brazil [158], allegedly presenting a more favorable environmental profile due to alleged lower toxicity to a range of non-target organisms [159], albeit its persistence in the marine environment with the potential for lethal and sublethal effects does occur in non-target organisms [160,161,162]. In addition, nanoparticle compositions of active substances such as teflubenzuron were claimed in the patent invention RU2406301C2 [116]. Nanoparticles of chlorfenapyr were developed to be more effective than conventional formulations by reducing environmental pesticide contamination and application costs, as their concentrations were one-fifth of those of the bulk counterpart. However, they exhibited similar performance [131]. In a complementary way, zein nanoparticles, proteins extracted from maize, considered an inexpensive, safe, and practical choice with which to produce nanoparticles, can be toxic to neonates of *Anticarsia gemmatalis* [66].

Concerning the nanoremediation of soybean chemical pesticides, the photodegradation under a Xe lamp of teflubenzuron and novaluron with a C3N4 nanofilm presented 82% and 69.4% degradation in 2 h, respectively [112]. The photodegradation of methomyl pesticide by ${TiO}_{2}$ doped with cadmium sulfate (${CdSO}_{4}$-doped ${TiO}_{2}$) nanoparticles under sunlight radiation presented a removal capacity of one gram of pesticide per gram of the introduced photocatalyst in one hour [163]. Assuming that thiodicarb degrades to methomyl immediately after spiking into a matrix of animal-derived food products, as stated in [164], the previous result must hold for thiodicarb. Photocatalytic processing with ${TiO}_{2}/{Na}_{2}S_{2}O_{8}$ (titanium dioxide/sodium persulfate) at the pilot plant under sunlight was performed in soil contaminated with chlorantraniliprole, imidacloprid, pirimicarb, and thiamethoxam, removing most of the insecticides and their main transformation products generated during the photoperiod [165]. The remediation of soil and water contaminated with chlorfenapyr was performed using iron and silver nanoparticles, achieving degradation results of up to 93.7% [166].

## 7. Environmental Risk and Human Health Considerations

Nanoformulations may alter exposure pathways (e.g., inhalable fine sprays, enhanced plant surface residence, and trophic transfer) relative to bulk formulations [42,43,49,76]. Risk assessment therefore requires (I) nano-specific physicochemical characterization under use conditions (size, charge, dissolution, corona, and release kinetics) [76,77,81]; (II) fate and transport studies on relevant Brazilian matrices (Oxisols, Ultisols, and tropical waters) [167,168,169]; (III) ecotoxicity panels spanning pollinators, aquatic invertebrates, soil biota, and vertebrate surrogates [79,80,170]; and (IV) human exposure scenarios for applicators, residents, and consumers (dietary residues) [49,171]. Human health data on actives such as imidacloprid (including reported fatal poisonings) underscore the need to ensure that nano-enabled delivery does not expand the systemic bioavailability or persistence of hazardous actives [133,134,172]. For polymeric carriers (e.g., star polycations), organismal studies report adverse outcomes at sublethal exposures, warranting the case-by-case evaluation of carrier toxicodynamics [121]. Absent a harmonized regulatory definition of “nanopesticide,” standard ecotoxicity protocols for bulk chemicals may be insufficient, motivating the development of adapted guidelines and nano-specific endpoints [41,42,49,81].

## 8. Photocatalytic Remediation and Environmental Fate and Cleanup of Pesticide Residues

While the prior sections addressed nanopesticides for on-plant pest suppression, photocatalysis, discussed here, concerns the post-application remediation of residues in water, soil, or processing streams, not direct in-field insecticidal action. Photocatalysis is a sustainable strategy consisting of the transformation of solar energy into chemical energy mediated by a photocatalyst for the mineralization of organic substances, ranging from different organic pollutants, such as pesticides, to living microorganisms, such as bacteria [173]. Emerging technologies employ nanomaterials as photocatalysts due to their remarkable performance in contrast to bulk counterparts [174], having large surface areas that provide more sites for reactants and photocatalyst interaction, resulting in accelerated reaction rates, tunable morphological and compositional properties, allowing for the optimization of photocatalytic performance, and the enhancement of reactivity as a result of intrinsic quantum confinement and surface-related effects, which avoid undesirable electron–hole recombination processes [175].

In such an eco-friendly approach, sunlight drives nanomaterials for redox/charge transfer processes from the absorption of visible and/or UV sunlight, enabled by the unique electronic structure of the nanophotocatalyst, which, in turn, starts a cascade of chemical reactions leading to the complete decomposition of organic substances into simple inorganic compounds such as CO_2_ and H_2_O, in a process known as mineralization [176].

**Figure 2:**
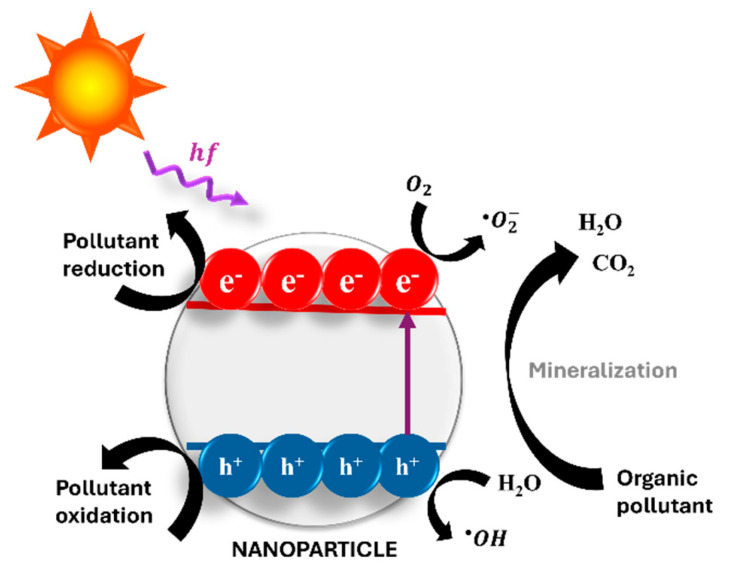
Representation of the solar photocatalytic degradation of different pollutants.

As the sunlight reaches the photocatalyst, electron–hole pairs are photogenerated and migrate to distinct regions of the photocatalyst’s surface, where electrons and holes simultaneously reduce and oxidize pollutants, respectively. Additionally, the photogenerated charge carriers on the surface of the photocatalyst can generate reactive oxygen species, which further degrade organic pollutants to mineralization [177]. A schematic picture of the solar photocatalytic degradation of different pollutants is depicted in Figure 2.

Beyond photocatalysis, several nano-enabled cleanup routes are gaining traction for pesticide removal in agricultural waters and processing streams: First, adsorption using high-area nanosorbents allows fast capture followed by easy recovery when magnets are built-in, including magnetic nanomodified activated carbon that removes thiamethoxam from sugarcane juice, enabling post-use separation and potential sorbent regeneration [124], and graphene oxide–chitosan–CuO hybrid particles that scavenged lambda-cyhalothrin and thiamethoxam from wastewater through combined π-π, hydrogen bonding, and metal-assisted interactions [122]. Prussian blue analogs provide another low-energy route: green-synthesized metal hexacyanoferrate nanoparticles captured chlorpyrifos, thiamethoxam, and tebuconazole with high removal efficiencies, illustrating how ion exchange and cage-like coordination sites can target diverse chemistries [123].

Second, catalytic reduction with reactive nanometals and bimetallic complements involves adsorption by chemically transforming priority pesticides into less toxic products. Successful cases for carbamates, organophosphates, and pyrethroids using robust nanomaterials that operate at ambient conditions can be seen in [178,179,180,181]. Third, nano-enabled separations deploy adsorptive or nanocomposite membranes that couple size exclusion with surface affinity to strip pesticides from complex matrices, offering inline polishing options for packing houses and agro-industries [179].

Fourth, nano-assisted phytoremediation uses benign nanoparticles as solubility enhancers or rhizosphere boosters to increase plant uptake and degradation; chlorfenapyr removal was accelerated by a green nano-phytoremediation strategy that paired plant systems with nanomaterial additives, reducing residues in both soil and water [166]. These approaches are complementary, can be combined in tandem with head-to-head performance benchmarks, and are attractive where energy input and secondary waste must be minimized while enabling sorbent recovery, regeneration, and safe end-of-life management [178,179]. Table 2 summarizes several cleanup methods deploying nonparticulate materials.

## 9. Future Perspectives and Challenges for Nanopesticides in Crops

Translating nano-enabled crop protection from laboratories and greenhouses into farm-scale conditions will require coordinated work on efficacy, safety, manufacturability, and regulation. Below, we outline near-term opportunities and the main challenges.

Field translation and agronomic performance: Demonstrations need head-to-head comparisons against labeled standards at equal active ingredient loads, under tropical rainfall, UV, and dust conditions, with standardized endpoints and confidence intervals [42,76]. Promising delivery gains reported for star polymer carriers, solid nanodispersions, nanoemulsions, and unimolecule systems should be validated across soybean, sugarcane, coffee, and citrus, including persistence after wash-off, canopy coverage, and residue decline curves [35,44,50,63,64,71]. IPM-compatible tools such as electrospun pheromone fibers and functional nano-dispensers merit farm-level trapping and disruption trials in Brazilian landscapes with attention to lure longevity and trap density [58,59,60,74].

Safe-by-design carriers and case-by-case assessment: Carrier chemistry can shift hazard independent of size. Differences in toxicity between micro- and nano-capsules have been linked to capsule composition, which supports case-by-case evaluation rather than a generic nano penalty or nano credit [78]. Cationic star polymer systems that improve plant uptake also showed organism-level effects in model species, which argues for carrier hazard banding and substitution if needed [45,121]. Pollinator panels and sublethal endpoints should be routine for nanoformulations of neonicotinoids and diamides used in citrus and sugarcane [80].

Exposure, fate, and matrices specific to a location: Nanoformulations can alter sorption, mobility, and durability relative to conventional products [43,76]. Fate studies on Oxisols and Ultisols and tropical waters are a priority, leveraging existing knowledge on pesticide leaching and sorption in these matrices [167,168,169]. Soil invertebrate tests and trophic transfer studies should reflect Brazilian edaphoclimatic conditions and community composition [79,170]. Physicochemical data must be generated under use conditions, including size distributions, surface charge, corona, and release kinetics, with good practice for zeta potential reporting [77,81].

Resistance management and IPM integration: Nano-delivery does not change molecular targets, which means resistance risk remains unless exposure profiles and spatial placement are managed [35,62]. Field programs should pair nanoformulations with refuges, rotate modes of action, and exploit spatial tools, such as pheromone fibers for mating disruption and attract-and-kill, to reduce selection pressure [58,59,149,150].

Manufacturing, green chemistry, and scalability: Scaling polymeric and hybrid systems requires reproducible particle size, loading, and release, with low-solvent footprints and robust shelf life under tropical storage. Green synthesis using botanical or microbial reductants is attractive but needs quality control, batch-to-batch consistency, and a toxicology that covers residual biochemicals and by-products [182,183,184]. Life cycle assessments should compare nano and non-nano options for energy, water, emissions, and waste.

Regulatory clarity and data standards: The absence of a harmonized legal definition of nanopesticides complicates registration pathways and post-market surveillance [41]. The EFSA technical requirements for small particles and nano risk assessment offer a structured model for data packages, including dissolution, transformation, and particle characterization, that could be adapted in Brazil [49,81,171]. Registrations should require reporting templates that link characterization and release data to exposure models and field efficacy, which will streamline cross-study comparisons [42,76].

Human and environmental safety prioritization: For actives with known clinical concerns, such as imidacloprid and thiamethoxam, nano-delivery must not expand systemic bioavailability or extend persistence in ways that elevate risk for applicators, residents, or consumers [80,133,134,172]. Dietary exposure models should incorporate nano-specific residue data and degradation kinetics.

Coupling with remediation: Where legacy residues or off-target deposition persist, coupling nano-delivery in the field with nano-enabled remediation in processing water or drainage, such as photocatalytic or adsorptive removal, can lower overall system risk [103,165,166,178,180]. This should remain a downstream safety measure rather than a justification for higher application rates.

Adoption and capacity building: Successful deployment will depend on supply chains that can deliver stable formulations, training on mixing and spraying, compatibility with existing equipment, and clear on-label instructions for handling potential nano aerosols [42]. Open data on performance and safety will support grower confidence and social license.

## 10. Final Remarks

Each year, Brazil consolidates itself as the largest agricultural exporter in the world [185]. The country, currently the leader in the export of coffee, sugarcane, soybeans, and oranges, became the world’s largest exporter of corn in 2023 [186]. However, the increase in productivity in Brazilian agriculture is also due to the application of agrochemicals [187]. Brazil is the world leader in the use of pesticides, with an average of 5 L per year per person [188,189]. Brazilian legislation on pesticide registration and commercialization is relatively less restrictive than in the European Union: 30% of pesticides used in the country are banned in the European Union [99,157,190].

Table 3 presents a comparison between conventional treatments and approaches using nanostructures for the main pests and diseases affecting coffee, sugarcane, orange, and soybean crops in Brazil. Nanotechnology is an alleged promising innovation to promote sustainable agriculture [191,192]. As a remake of the evolution of technologies on pesticides, nano-sized technologies for agrochemicals have started with inorganic nanoparticles [70,193], synthetic organic compounds by nanosizing hazardous/banned pesticides [78,194], and lower-risk synthetic organic compounds by nanosizing pesticides with more favorable environmental profile due to alleged lower toxicity to a range of non-target organisms and/or hybrid technologies [72,195,196]. In an attempt to make pesticide nanotechnology more environmentally friendly, green nanosynthesis, i.e., the synthesis of nanostructures by botanical or microbial compounds, is a growing field of interest for researchers and the pesticide industry. It is hypothesized that biologically derived compounds allow better target specificity, performing eco-safe behavior against non-target organisms [182,183,184,197]. To enhance pesticide activity with minimal environmental risks, also preventing pesticide loss and improving utilization efficiency, controlled-release pesticide systems aim to achieve pesticide release in a spatial- and temporal-controlled manner [35,62,73].

Lastly, pesticide removal is imperative due to their high persistence, toxicity, and potential for bioaccumulation. Nanotechnological methods, including adsorption and degradation through photocatalysis and catalytic reduction, offer a promising strategy for eliminating pesticide pollution due to their economic, rapid, and highly efficient approach. Indeed, the nanoparticles display a unique surface area and surface activity, fundamental to the catalytic reactions used to degrade pesticides into benign products [123,178,179,180,181,198]. Undoubtedly, nanotechnology will be essential to a sustainable future in crop protection, playing a vital role in food production. However, it becomes mandatory to ensure that its application in pest control does not result in harm to the environment and humans.

## Figures and Tables

**Table 1:** Nano-enabled pest control in crops.

Crop	Target Pest or Objective	Nano System/Formulation	Intended Function	Reported Effect	Proposed Mechanism *	Refs.
Coffee	Coffee berry borer *Hypothenemus hampei*	ZnO nanoparticles	Direct bioactivity	Sublethal and adverse biological effects in lab assays	Membrane disruption and ROS generation typical of metal oxide NPs	[47,48,69]
Coffee	Coffee berry borer *H. hampei*	CuO nanoparticles	Direct bioactivity	As above, dose-dependent effects on biological traits	Membrane damage, oxidative stress, and ionic release	[47,48,69]
Coffee	Coffee berry borer *H. hampei*	CeO_2_ nanoparticles	Direct bioactivity	Adverse effects on biological characteristics	Redox imbalance and enzyme perturbation at nano–bio interface	[69]
Citrus	Vector *Diaphorina citri*	Thiamethoxam nanoformulation (encapsulation and release control)	Delivery optimization	Effective psyllid management with improved encapsulation and profiling	Improved leaf wetting and retention, controlled release, and enhanced uptake	[65,82]
Citrus	Vector *D. citri*	Functional nano-dispenser for imidacloprid	Spatial targeting	Vector suppression with point-source release	Sustained, localized release from nano-dispenser matrices	[74]
Citrus	Medfly *Ceratitis capitata*	Electrospun nanofibers loaded with trimedlure	Attract-and-kill, monitoring	Longer-lasting lure release, improved trap performance	Diffusion-controlled semiochemical release from nanofibers	[75]
Citrus	Medfly (alternative)	Magnetite nanoparticles (trimedlure-free concept)	Attract-and-kill or toxic contact	Dose-dependent larval lethality in the lab	ROS-mediated stress and membrane interactions of Fe oxide NPs	[83]
Sugarcane	Sugarcane borer *Diatraea saccharalis*	Novaluron nanoparticles	Delivery optimization of IGR	Comparable bioactivity to commercial formulations with potential dose economy	Better dispersion and controlled release from nano-capsules	[84]
Soybean	Fall armyworm *Spodoptera frugiperda*	ZnO nanoparticles	Direct bioactivity	Larval mortality, deformities, reduced fecundity, and hatch	Membrane disruption, ROS generation, ionic release	[48,67]
Soybean	Fall armyworm *S. frugiperda*	Copper-based nanoparticles	Direct bioactivity	Strong larvicidal and antifeedant activity; immune effects	Oxidative stress, membrane interactions	[68]
Soybean	Fall armyworm *S. frugiperda*	Mixed NPs (Cu, KI, Ag, and Bd)	Direct bioactivity	Significant insecticidal effects in the lab; soil impact assessed	Multi-modal surface reactivity and redox stress	[85]
Soybean	Velvetbean caterpillar *Anticarsia gemmatalis*	Zein protein nanoparticles	Carrier with intrinsic bioactivity	Direct insecticidal activity: mechanistic lesions documented	Contact toxicity and gut interaction of protein-based NPs	[66]
Various crops	Multiple targets	Chlorantraniliprole solid nanodispersions	Stabilize and improve the dispersion of poorly soluble AI	Retained potency after high-pressure homogenization; improved handling	Enhanced dissolution, better leaf coverage, and uptake	[64]
Field settings	Multiple pathosystems	Unimolecule nanopesticide delivery system	Field-scale delivery optimization	Improved field control across systems	Increased deposition and penetration from nano-architecture	[50]
Model: aphid	Green peach aphid *Myzus persicae*	Thiamethoxam complexed with star polycation (SPc)	Foliar adhesion and plant uptake boost	Higher contact and stomach toxicity at the same AI load	Improved wetting, retention, and plant uptake via cationic carrier	[45,63]
Botanicals	Soft-bodied pests, general	Plant oil nanoemulsions	Stabilize botanicals, enhance coverage	Improved control outcomes and dose economy vs. coarse emulsions	Better dispersion, smaller droplets, improved cuticular penetration	[55,71]
Tomato	The tomato leafminer, *Tuta absoluta*	Nano-silica gel	Nanodelivery systems for pesticides	Nano-silica gel significantly increased the weight of the harvested tomato crop (Kg/feddan) compared to silica gel and the control	Dissolution, biodegradation, diffusion, and osmotic pressure at a specific pH	[86]
Strawberry	*Botrytis cinerea* Pers	Nanosized silver-chitosan	Inhibit the growth of B. cinerea and prevent gray mold decay	Strawberry coated with nano Ag-IrCTS: Showed no signs of infection for 4 days. By the end of the 7-day storage period, fungal decay appeared in just 10% of strawberries	Membrane disruption, ROS generation, and ionic release	[87]
IPM dispensers	*Spodoptera litura* and tree-fruit moths	Electrospun pheromone nanofibers	Mating disruption or mass trapping	Sustained release with strong trapping performance	Diffusion-controlled release from nanofibers	[58,59]

* at the pest or plant interface. AI = active ingredient.

**Table 2:** Nano-enabled remediation of pesticide residues.

Matrix or Context	Target Pesticide(s)	Nano Material or System	Reported Effect	Mechanism	Ref.
Water, soil	Chlorpyrifos	Cu nanoparticles under natural daylight	Photocatalytic mineralization of chlorpyrifos	Visible-light photocatalysis on Cu/Cu_2_O surfaces	[103]
Water	Chlorpyrifos	g-C_3_N_4_/TiO_2_ nanocomposite	Photodegradation with identified reactive species	Heterojunction-enabled charge separation and ROS production	[137]
Soil, aquatic tests	Novaluron; Teflubenzuron	C_3_N_4_ nanofilm (visible light)	64 percent novaluron and 82 percent teflubenzuron degraded in 2 h	Photocatalytic oxidation via graphitic carbon nitride	[112]
Water	Methomyl	CdSO_4_-doped TiO_2_ nanoparticles	Fast removal with high capacity under sunlight	Doped TiO_2_ photocatalysis with enhanced charge separation	[163]
Field soil and irrigation water	Chlorantraniliprole, imidacloprid, pirimicarb, and thiamethoxam	TiO_2_/Na_2_S_2_O_8_, pilot-scale, sunlight	Removal of most parents and main transformation products	Photocatalysis with persulfate oxidation	[165]
Soil and water	Chlorfenapyr	Fe and Ag nanoparticles	Up to 93.7 percent degradation	Nano-catalyzed reduction and oxidative pathways	[166]
Sugarcane juice, water	Thiamethoxam	Magnetic nanomodified activated carbon	Efficient removal from juice and water	High-area adsorption with magnetic separation	[124]
Water	Thiamethoxam, chlorpyrifos, tebuconazole	Green-synthesized metal hexacyanoferrate NPs	70 to 98 percent solar degradation of thiamethoxam; broad removal	Photocatalysis and adsorption on Prussian blue analogs	[123]
Water	Endosulfan	Cu/Cu_2_O core–shell nanoparticles	Mineralization under light	Plasmonic–semiconductor photocatalysis at the interface	[96]

**Table 3:** Comparison of conventional and nanostructure-based treatments for pests and diseases in Brazilian crops.

Crop	Main Disease/Pest	Conventional Agrochemical Strategy	Nanoparticulate Treatment (s)
Coffee	Coffee berry borer	Endosulfan (banned since 2013)	Zinc oxide (ZnO), copper oxide (CuO), and cerium oxide (CeO_2_) nanoparticles
	Coffee leaf miner	Chlorpyrifos (still prevalent)	Titanium dioxide (TiO_2_) nanoparticles (UV light), copper nanoparticles (natural daylight) for photodegradation
Sugarcane	Sugarcane borer	Novaluron	Novaluron nanoparticles, carbon nitride nanofilm for photodegradation
	Sugarcane weevil	Thiamethoxam	Nanometerization of thiamethoxam by polymers, nano-sized thiamethoxam/star polycation complexes
			Metal hexacyanoferrate nanoparticles, magnetic nanomodified activated carbon for removal
Orange	Citrus greening disease	Control of the vector: thiamethoxam, imidacloprid	Nanosized thiamethoxam, nano-dispenser strategy for imidacloprid, and imidacloprid nanoparticles
	Mediterranean fruit fly (Medfly)	Trimedlure-baited traps	Nanofiber formulations containing trimedlure, magnetite nanoparticles (trimedlure-free)
Soybean	Fall armyworm	Thiodicarb, methomyl, chlorantraniliprole, and flubendiamide (not all registered)	Cu, KI, Ag, and Bd nanoparticles, commercial zinc oxide nanoparticles, copper oxide nanoparticles, chlorantraniliprole nanoparticles, and functionalized thiodicarb nanoparticles
	Velvetbean caterpillar	Benzoylphenylureas (novaluron, teflubenzuron), chlorfenapyr	Nanoparticle compositions of teflubenzuron, nanoparticles of chlorfenapyr, and zein nanoparticles
	Pesticide residues (general)	Various conventional applications	C_3_N_4_ nanofilm for the photodegradation of teflubenzuron and novaluron, CdSO_4_-doped TiO_2_ nanoparticles for methomyl (and potentially thiodicarb), TiO_2_/Na_2_S_2_O_8_ for chlorantraniliprole, imidacloprid, pirimicarb, and thiamethoxam, and iron and silver nanoparticles for chlorfenapyr
